# Xue-fu-Zhu-Yu decoction protects rats against retinal ischemia by downregulation of HIF-1α and VEGF via inhibition of RBP2 and PKM2

**DOI:** 10.1186/s12906-017-1857-2

**Published:** 2017-07-14

**Authors:** Shu-Qiu Tan, Xue Geng, Jorn-Hon Liu, Wynn Hwai-Tzong Pan, Li-Xiang Wang, Hui-Kang Liu, Lei Hu, Hsiao-Ming Chao

**Affiliations:** 1 Department of Ophthalmology, Affiliated Hospital of Taishan Medical University, Taishan, Shandong China; 2 Department of Pharmacology, School of Medicine, Shandong University, Jinan, Shandong China; 3 Department of Ophthalmology, Cheng Hsin General Hospital, Taipei, Taiwan; 4 Institute of Pharmacology, School of Medicine, National Yang-Ming University, Taipei, Taiwan; 5 Division of Basic Chinese Medicine, National Research Institute of Chinese Medicine, Ministry of Health and Welfare, Taipei, Taiwan; 6 Department of Chinese Medicine, School of Chinese Medicine, China Medical University, Taichung, Taiwan

## Abstract

**Background:**

Retinal ischemia-related eye diseases result in visual dysfunction. This study investigates the protective effects and mechanisms of Xue-Fu-Zhu-Yu decoction (XFZYD) with respect to retinal ischemia.

**Methods:**

Retinal ischemia (I) was induced in Wistar rats by a high intraocular pressure (HIOP) of 120 mmHg for 1 h, which was followed by reperfusion of the ischemic eye; the fellow untreated eye acted as a control. Electroretinogram (ERG), biochemistry and histopathology investigations were performed.

**Results:**

Significant ischemic changes occurred after ischemia including decreased ERG b-wave ratios, less numerous retinal ganglion cells (RGCs), reduced inner retinal thickness, fewer choline acetyltransferase (ChAT) labeled amacrine cell bodies, increased glial fibrillary acidic protein (GFAP) immunoreactivity and increased vimentin Müller immunolabeling. These were accompanied by significant increases in the mRNA/protein concentrations of vascular endothelium growth factor, hypoxia-inducible factor-1α, pyruvate kinase M2 and retinoblastoma-binding protein 2. The ischemic changes were concentration-dependently and significantly altered when XFZYD was given for seven consecutive days before or after retina ischemia, compared to vehicle. These alterations included enhanced ERG b-wave amplitudes, more numerous RGCs, enhanced inner retinal thickness, a greater number of ChAT immunolabeled amacrine cell bodies and decreased GFAP/vimentin immunoreactivity. Furthermore, decreased mRNA levels of VEGF, HIF-1α, PKM2, and RBP2 were also found. Reduced protein concentrations of VEGF, HIF-1α, PKM2, and RBP2 were also demonstrated. Furthermore, there was an inhibition of the ischemia-associated increased ratios (target protein/β-actin) in the protein levels of VEGF, HIF-1α, PKM2, and RBP2, which were induced by Shikonin, JIB-04 or Avastin.

**Conclusion:**

XFZYD would seem to protect against well-known retinal ischemic changes via a synergistic inhibition of RBP2 and PKM2, as well as down-regulation of HIF-1α and a reduction in VEGF secretion.

**Electronic supplementary material:**

The online version of this article (doi:10.1186/s12906-017-1857-2) contains supplementary material, which is available to authorized users.

## Background

Retinal ischemia related diseases include central retinal artery occlusion (CRAO), central retinal vein occlusion (CRVO), branch retinal artery occlusion (BRAO), branch retinal vein occlusion (BRVO), glaucoma, and age-related macular degeneration (AMD) [1–3]. All of these disorders can result in serious complications and thus the treatment of retinal ischemia is vital to patient outcomes.

Glutamate receptor rich retinal ganglion cells (RGCs) and amacrine cells, and their neuronal fibers are situated in the inner retina, which makes them susceptible to ischemia/reperfusion (I/R) injury [1, 3]. After I/R, enhanced vimentin immunoreactivity in Müller cells can be seen [4]. Retinal or choroidal ischemia associated angiogenesis [5] often results in subretinal fluid and bleeding, which in turn leads to visual dysfunction. It is thus important to discover novel approaches that are able to stimulate self-protective mechanisms within the eye and that help to avoid detrimental neovascularization. Elevated concentrations of the hypoxia-inducible factor-1α (HIF-1α) have been demonstrated after retinal ischemia [6]. HIF-1α interacts with hypoxia dependence components of various hypoxia related genes and up-regulates the level of vascular endothelium growth factor (VEGF) [6]. Liu et al. [7] have also shown that oxidative stress in the human retinal pigment epithelium leads to VEGF up-regulation. All the above results support the hypothesis that up-regulation of HIF-1α and VEGF in the retina is directly related to ischemic insult. The KDM5/JARID1 family of Fe(II)- and α-ketoglutarate-dependent demethylases remove methyl groups from tri- and dimethylated lysine 4 of histone H3. Figure 2c of Wang et al. [8] demonstrated that purified Jumonji demethylases representing several subfamilies exhibited distinct sensitivities to JIB-04 in vitro, with JARID1A (KDM5A, also known as retinoblastoma-binding protein 2, RBP2) being the most sensitive (IC50 = 230 nM). Other proteins include JMJD3 (KDM6B) and JMJD2C (KDM4C), which were more resistant (IC50 ~ 1 μM) to JIB-04. Presently, JIB-04, a paninhibitor of the Jumonji demethylase superfamily, was thus selected to observe the inhibition of RBP2 upregulation in retinal ischemia. Pyruvate kinase (PK), glycolytic enzyme, catalytically convert phosphoenolpyruvate into pyruvate, generating one molecule of ATP. In addition, the PKM2 inhibitor shikonin [9], and the anti-VEGF agent avastin was presently selected to observe the inhibition of PKM2 and/or VEGF overexpression after retinal ischemic insult. An investigation as to whether an overexpression of VEGF, HIF-1α, PKM2 and RBP2 coexists in the ischemic retina has also been included in the present investigation.

Xue-Fu-Zhu-Yu decoction (XFZYD) is a famous traditional Chinese medicine formula that has been used to manage with cardiovascular disorders for several centuries [10]. The study of Lee et al. [11] suggested that XFZYD increased the potential of recombinant tissue plasminogen activator (tPA) as a neuroprotection agent against rat brain ischemia. The report of Zhou et al. [12] has also indicated that XFZYD seems to be able to alleviate hypoxia and protect liver sinusoidal endothelial cell function and does this by decreasing the levels of VEGF and HIF-1α. The formulation of XFZYD consists of eleven components (with respective representative active compounds in the bracket), namely *Radix Angelicae Sinensis* (Dang Gui: ferulic acid) [13, 14], *Radix Rehmanniae glutinosae* (Di Huang: catalpol) [15], *Radix Paeoniae Rubra* (Chi Shao: peoniflorin) [16], *Rhizoma Ligustici Chuanxiong* (Chuan Xiong: ferulic acid) [13, 14], *Semen Pruni Persicae* (Táo Rén: amygdalin) [17], *Flos Carthami Tinctorii* (Hong Hua: hydroxysafflor yellow A, HSYA) [18], *Radix Glycyrrhizae Uralensis* (Gan Cao: glycyrrhizin) [19], *Fructus Auranti* (Zhi Qiao: costunolide) [20], *Radix Bupleuri Chinense* (Chai Hu: saikosaponin) [21], *Radix Achyranthis Bidentatae* (Niu Xi: *Achyranthes bidentata* polysaccharides) [22], and *Radix Platycodi Grandiflori* (Jie Geng: platycodins) [23]. The important known effects of the defined active compounds described above are as follows. Ferulic acid has been proved to scavenge hydroxyl radical and is able to provide neuroprotection against retinal ischemia [13, 14]. Catalpol and the *Achyranthes bidentata* polysaccharides have antioxidative potential [15, 22]. Peoniflorin [16], amygdalin [17], HSYA [18], glycyrrhizin [19], costunolide [20] and saikosaponin [21] are able to protect against brain ischemia. Furthermore, amygdalin and HSYA have been shown to act additively by decreasing plasma viscosity and platelet aggregation. Aqueous extract from *Platycodon grandiflorum such as* platycodins [23] has a significant hepatoprotective effect and acts by reducing nitric oxide and counteracting lipid peroxidation dose-dependently.

The purpose of the current research is to investigate if XFZYD is able to alleviate retinal ischemic insult and to explore the decoction’s influences on electroretinogram (ERG), the density of RGCs, retinal thickness, choline acetyltransferase (ChAT) immunopositive amacrine cells, and glial fibrillary acidic protein (GFAP) immunolabelling of Müller cells and vimentin immunolabelling of Müller cells. In addition, the mRNA and protein concentrations of VEGF, HIF-1α, PKM2 and RBP2 were analyzed by real-time polymerase chain reaction (rtPCR) and Western blot, respectively, in the presence/absence of XFZYD.

## Methods

### Animals

Animals were utilized based on the Association for Research in Vision and Ophthalmology Statement for the Use of Animals in Ophthalmology and Vision Research, and all the experiments were agreed to the Institutional Animal Care and Use Committee at Cheng Hsin General Hospital (CHGH; Taipei, Taiwan; Approval No: CHIACUC 104–05). Six-week-old male Wistar rats (BioLasco, Taipei, Taiwan) were bred in a large plastic cage (Shineteh Instruments Co., Ltd., Taipei, Taiwan) containing fewer than six rats at a humidity of 40–60% and at 19–23 °C. The rats (*n* = 115) were arbitrarily allocated into one of the following groups: Normal (*n* = 12), Sham (*n* = 16–1 = 15; one rat died during flurogold retrograde labeling), Vehicle + I/R (*n* = 16), XFZYD_1.35_ + I/R (*n* = 16), XFZYD_2.7_ + I/R (*n* = 16), I/R + Vehicle (*n* = 5), I/R + XFZYD_1.35_ (*n* = 6), I/R + XFZYD_2.7_ (*n* = 16), and I/R + Inhibitor (Shikonin, *n* = 4; JIB-04, *n* = 4–1 = 3: one JIB-04 blot was ill-defined; Avastin, *n* = 4). The number of the rats used in this study totaled 120, including 5 animals that died during the induction of pressure-induced retinal ischemia (Additional file 1). All rats were kept on a 12-h light/dark cycle with 12–15 air changes per hour. The rats received food and water ad libitum. The rat experiments were carried out in a manner that followed the *Animal* Research: Reporting of In Vivo Experiments (ARRIVE) guidelines.

### Animal anesthesia and euthanasia

Anesthesia was carried out using 100 mg/kg ketamine (Pfizer, NY, USA) and 5 mg/kg xylazine (Sigma-Aldrich, MO, USA), which were injected intraperitoneally into the rats. An intraperitoneal (i.p.) injection of at least 140 mg/kg sodium pentobarbital (SCI Pharmtech, Taoyuan, Taiwan) was given to sacrifice the rats in a considerate and painless manner (Scientific Procedures Act 1986).

### Retinal ischemia induction

The rats (200–250 g) were constrained in a stereotaxic frame under i.p. anesthesia induced by the defined doses of ketamine and xylazine. A high intraocular pressure (HIOP) of up to 120 mmHg for 60 min was induced by cannulating the anterior chamber of the ischemic eye with a 30-gauge needle attached to a raised normal saline bottle. The detection of a pale retina indicated the establishment of retinal ischemia [1, 2, 24]. As a sham control, one eye of a control rat was given the same procedure without elevating the saline bottle [2].

### Drug provision

An intake of 1.35 or 2.7 g/kg/day XFZYD (Sun Ten Pharmaceutical CO, Taichung, Taiwan) was administered for seven continuous days before or after HIOP induced retinal ischemia and the rats were then sacrificed. The test rat’s eye that received ischemia was provided with a constant quantity (4 ml) of XFZYD or the “same” volume of vehicle.

After anesthesia as described, the rats’ pupils were dilated with 1% tropicamide (Alcon, ZG, Switzerland) and 2.5% phenylephrine (Akorn, Inc., IL, USA); in addition there was anesthesia of the ocular surface with 0.5% proparacaine (Alcon, ZG, Switzerland); then, a 30-gauge needle attached to a 25 μl syringe was used to perform the intravitreal injections. In certain instances, intravitreal injections (5 μl) of 4 μM Shikonin (Sigma-Aldrich, MO, USA), of 10 μM JIB-04 (Sigma-Aldrich, MO, USA), of 100 mg/4 ml Avastin (Hoffmann-La Roche, Basel, Switzerland) or of vehicle (an equal volume of dimethyl sulfoxide; J.T.Baker, NJ, USA) were given to ischemic eyes for fifteen minutes and retinal ischemia was induced by HIOP later.

### Flash ERG measurement

Flash ERG was recorded on all animals before retinal ischemia (day 0) and after seven consecutive days of preischemia or postischemia administration of XFZYD or vehicle. The rats were kept in the dark for approximately 8 h, then they were given mentioned anesthetics before the ERG measurements; for this, the pupils were dilated with 1% tropicamide and 2.5% phenylephrine (Akorn, Inc., IL, USA) as well as the ocular surface anesthetized with 0.5% proparacaine (Alcon, ZG, Switzerland). A light source was put 2 cm before the rat eye to achieve a stimulation of 0.5 Hz. Fifteen continuous recordings were collected at 2-s intervals and at 10 kHz; the amplification and the average of the responses were recorded using an amplifier P511/regulated power supply 107/stimulator PS22 (Grass-Telefactor; AstroNova, QC, Canada). On a comparative basis, the b-wave quotient, i.e., the b-wave amplitude of the treated ischemic eye divided by the b-wave amplitude of the untreated fellow normal eye, was measured [1, 3]. The animals with the b-wave ratios above 125% and below 75% were excluded.

### Measurement of fluorogold retrograde labeling RGCs

Using the above anesthetics, a 2-cm incision was made in the rat’s scalp and two small holes drilled in the skull as described previously [2]. Next, 5% fluorogold (2 μl; Sigma-Aldrich, MO, USA) were given by a microinjector at locations of 3.8, 4.0, and 4.2 mm down the skull. In all groups, the fluorogold injection was performed three days before the rats were sacrificed.

After sacrifice, retinal tissue was carefully isolated, incubated with 4% paraformaldehyde fixative, sectioned and processed as described previously [2]. The definition of mean RGC density was the total RGC amount divided by the whole retinal area [2].

### Cresyl violet staining

The animals were sacrificed and injected with 0.9% saline (*w*/*v*) into the cardiac cavity. Next, the eyeballs were enucleated, fixated with 4% (*w*/*v*) paraformaldehyde and embedded in paraffin (Tissue-Tek TEC 5; Sakura, Alphen aan den Rijn, Netherlands), then processed for sectioning (5 μm). The sectioned retinal pieces were labeled with cresyl violet and evaluated under a light microscope (Leica, Heidelberg, Germany). Afterwards, the sectioned retinal pieces were photographed under the microscope with an identical magnifying power (Ilford Pan-F plus film, 50 ASA). Retinal layer thicknesses were calculated from the photographs.

To quantify the degree of retinal ischemic injury, we measured the whole retinal thicknesses [from the inner limiting membrane (ILM) to the retinal pigment epithelium (RPE) layer] and the inner retinal thicknesses [(from the ILM to the inner nuclear layer (INL)]. All measurements were carried out 1 mm from the optic nerve head. Three consecutive sectioned retinal pieces for each eye were measured to obtain a mean value. The laboratory researcher carrying out the measurements was masked to all information on the retinal sections when evaluating the changes in the thickness among the five groups, namely Sham, Vehicle + I/R, XFZYD_1.35_ + I/R, XFZYD_2.7_ + I/R, I/R + XFZYD_2.7_.

### Immunofluorescence analysis

After sacrifice, the eyeballs were prepared in paraffin and processed for retinal sections described above. Next, the sectioned retinal pieces were immunohistochemically processed utilizing the following primary antibodies: goat anti-ChAT polyclonal antibody (Millipore, CA, USA), rabbit anti-GFAP polyclonal antibody (Millipore, CA, USA) and mouse anti-vimentin monoclonal antibody (Sigma-Aldrich, MO, USA). Thereafter, the retinal sections were soaked in the appropriate secondary antibodies: rhodamine-bound rabbit anti-goat antibody (Millipore, CA, USA), fluorescein isothiocyanate (FITC)-bound goat anti-rabbit IgG (Millipore, CA, USA) or FITC-bound goat anti-mouse IgG (Millipore, CA, USA). Simultaneously, the cellular nucleus was labeled with 4,6-diamidine-2-phenylindole dihydrochloride (DAPI; EMD Chemicals, Darmstadt, Germany) as described previously [1, 24]. A fluorescence microscope was used to examine the sectioned retinal pieces as described previously [1, 24]. To grade the immunoreactivity levels in the retinal sections among the various experimental groups, a researcher who was blinded to the status of the sections was requested to compare the immunoreactivity level of the various experimental groups against the sham group (control).

### Measurement of the concentrations of various mRNAs in the retina by rtPCR

The mRNA concentrations of retinal VEGF, HIF-1α, PKM2 and RBP2 were measured by rtPCR [3, 25]. One day after a retinal ischemic insult and pre−/post-ischemia administration of the various compounds or after a sham process, the animals were sacrificed and the retinal tissues were dissected. Ultrasound emulsification was then performed in the presence of Tri Reagent (Sigma, Missouri, USA). Isolation of retinal RNA was carried out and first strand complementary DNA (cDNA) was synthesized after 2 μg RQ1 RNase-Free DNase (0.05 U/μl; Promega)-incubation utilizing High Capacity RNA-to-cDNA Master Mix (Applied Biosystems, MA, USA). Afterwards, the first-strand cDNA went through rtPCR; Fast Smart Quant Green Master Mix (Bio-protech, Gangwon-do, Korea) was used. The PCR was started by maintaining at 95 °C for 20 s and, subsequently, 40 rounds of 95 °C for 3 s and 60 °C for 30 s were carried out. Cycling was performed on a StepOne Plus™ Real-Time PCR System (Applied Biosystems, MA, USA). Measurement of the relative level of a target mRNA (a comparative assay) was carried out utilizing the cytoskeleton gene β-actin as the internal control. This procedure allowed the measurement of the normalized level of the target mRNA and considers the differences in the quantity of total mRNA applied to each reaction (ΔCt = Ct target-Ct β-actin; cycle threshold, Ct). The relative VEGF/HIF-1α/PKM2/RBP2 level differences caused by ischemia or the sham process were measured as fold variations relative to the contralateral untreated control normal retina using the calibration equation (Ct = ΔCt induced-ΔCt normal). Relative gene expression was measured using the 2^-ΔΔCt^ equation, as mentioned in the company procedures [3, 25]. The results retrieved for each experimental procedure were collected together, and a total ratio difference related with the Sham (control) was calculated. Table 1 describes the PCR oligonucleotide primers used for the rtPCR (Mission Biotech Co. Ltd., Taipei, Taiwan).

**Table 1:** Sequences of oligonucleotide primers and details of polymerase chain reactions

mRNA	Primers (5’→3’) F: Forward R: Reverse	Bases in base pairs	Product size	Cycles profile Denaturation/annealing/extension (Temperature and time in seconds)	Cycles number
β-actin	F: AGGGAAATCGTGCGTGACAT	694 713	150	95°C / 95°C / 60°C	40
	R: GAACCGCTCATTGCCGATAG	824-843		(20s / 3s / 30s)	
VEGF	F: GCGGGCTGCTGCAATG	1262-1277	268	95°C / 95°C / 60°C	40
	R: TGCAACGCGAGTCTGTGTTT	1528-1547		(20s / 3s / 30s)	
HIF-1α	F: ACAGCTCCCCAGCATTTCAC	2748-2767	90	95°C / 95°C / 60°C	40
	R: GGACAAACTCCCTCACCAAAAA	2837-2816		(20s / 3s / 30s)	
PKM2	F: TCTACGTGGACGATGGGCT	833-851	403	95°C / 95°C / 60°C	40
	R: AGGAAGACCTTCTCTGCCGGA	1215-1235		(20s / 3s / 30s)	
RBP2	F: TTGTGGTGACGTTTCCTCGT	2093-2112	213	95°C / 95°C / 60°C	40
	R: CAGCCAGCCCCACATCTAAG	2305-2286		(20s / 3s / 30s)	

### Measurement of the protein levels by the western blot method

One day after a retinal ischemic insult and pre−/post-ischemia administration of the mentioned compounds or after a sham procedure, the animals were sacrificed. The retinal tissues were isolated and emulsified using ultrasound in lysis buffer, namely mammalian protein extraction reagent (HyCell, Taipei, Taiwan). The same quantities of denatured protein (100 μg/32 μl/well) were separated on a NuPAGE® Tris-Acetate mini gel electrophoresis system (Invitrogen, MA, USA) using a 3 ~ 8% separating gel and a 3.2% stacking gel as described previously [7, 25]. The separated proteins were transferred to a polyvinylidene difluoride (*PVDF*) membrane (Millipore, MA, USA). Next, the membranes were blocked with 5% fat-free skimmed milk (Fonterra, Taoyuan, Taiwan) in phosphate-buffered saline (PBS) for 1 h at room temperature.

The blots were then soaked one night at 4 °C in the following primary antibodies, namely, mouse monoclonal anti-β-actin antibody (1:80,000, Novusbio, MA, USA), mouse monoclonal anti-VEGF antibody (1:200, Novusbio, MA, USA), mouse monoclonal anti-HIF-1α antibody (1:1000, Abcom, Cambridge, UK), rabbit polyclonal anti-PKM2 antibody (1:600, Abcom, Cambridge, UK) and rabbit monoclonal anti-RBP2 antibody (1:500, Abcom, Cambridge, UK). Next, the blots were soaked in the appropriate secondary antibody, either horseradish peroxidase-bound goat anti-rabbit or horseradish peroxidase-bound goat anti-mouse IgG (1:2000 or 1:10,000, respectively, Santa Cruz Biotechnology, TX, USA) at 37 °C for 1 h. The primary/secondary antibodies were diluted in 5% fat-free skimmed milk. Finally, the membranes were developed using an enhanced chemiluminescent analysis system (HyCell, Taipei, Taiwan), which was followed by exposure to an X-ray film (Fujifilm, Tokyo, Japan). Analysis of the quantity of the each protein was carried out by scanning densitometry.

### Analytical statistics

The results are presented as the mean ± standard deviation (SD). One-way analysis of variance (ANOVA) followed by the Dunnett’s test was performed to compare the results. A *p* value of <0.05 was defined as statistical significance. Sigma Plot 12.5 (Systat Software, CA, USA) was used to analyze the data and draw the quantitative plots.

## Results

### The influence of XFZYD on ERG b-wave amplitude

**Fig. 1:**
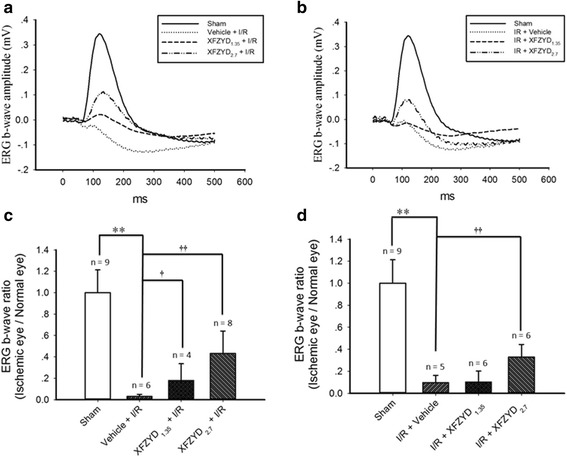
Electroretinogram (ERG): the influence of preischemia administration or postischemia administration of XFZYD on retinal ischemia. In comparison with the sham retina (Sham), the b-wave amplitude was considerably reduced after pressure-induced retinal ischemia plus reperfusion and preischemia administration (Vehicle + I/R; 1**a**) or postischemia administration of Vehicle (I/R + Vehicle; 1**b**) in a representative rat. This reduction was attenuated dose-dependently by preischemia administration (at 1.35 g/kg/day, XFZYD_1.35_ + I/R; at 2.7 g/kg/day, XFZYD_2.7_ + I/R, 1**a**) or postischemia administration of XFZYD (at 1.35 g/kg/day, I/R + XFZYD_1.35_; at 2.7 g/kg/day, I/R + XFZYD_2.7_, 1**b**) in one animal of each relevant group. As compared with the Sham group, a significant (**, *p* < 0.01) decrease in b-wave ratio was revealed for the Vehicle + I/R (1**c**) or I/R + Vehicle (1**d**) group following pressure-induced retinal ischemia. This ischemia-induced decrease was alleviated in a dose responsive and significant manner (†, *p* < 0.05; ††, *p* < 0.01, 1**c**) by the preischemia administration of 1.35 and 2.7 g/Kg/day of XFZYD. Postischemia administration of XFZYD at 2.7 g/Kg/day (1**d**) also significantly (††, *p* < 0.01) alleviated the reduction. The data are shown as mean values ± SD. XFZYD, Xue-Fu-Zhu-Yu decoction

In the retina subjected to the sham procedure (Sham, Fig. 1), the ERG b-wave amplitude was measured as 0.36 mV. After retinal I/R, the b-wave amplitude was reduced to a considerable extent. The reduction was not affected by preischemia or postischemia administration of vehicle (Vehicle + I/R: 0.01 mV, Fig. 1a; I/R + Vehicle: 0.02 mV, Fig. 1b). Nevertheless, preischemia XFZYD administration (XFZYD_1.35_ + I/R, 1.35 g/kg/day; XFZYD_2.7_ + I/R, 2.7 g/kg/day, Fig. 1a) and postischemia XFZYD administration (I/R + XFZYD_1.35_, 1.35 g/kg/day; I/R + XFZYD_2.7_, 2.7 g/kg/day, Fig. 1b) mitigated the ischemia-associated reduction in b-wave and the amplitudes were elevated to 0.03, 0.13, 0.02 and 0.10 mV, respectively.

As shown in Fig. 1c (*n* = 4 ~ 9), as compared to the sham group (1.00 ± 0.21), the b-wave ratio was significantly (*p* < 0.001) reduced in the Vehicle + I/R group (0.03 ± 0.02). Of note, after preischemia XFZYD administration, a dose-responsive (1.35 vs. 2.7 g/kg/day) and significant (at 1.35 g/kg/day, *p* = 0.04; at 2.7 g/kg/day, *p* < 0.001) mitigation was detected in the ischemia-induced b-wave ratio reduction after I/R (XFZYD_1.35_ + I/R vs. XFZYD_2.7_ + I/*R* = 0.18 ± 0.16 vs. 0.43 ± 0.21).

Again, in Fig. 1d (*n* = 5 ~ 9), as compared to the sham group (1.00 ± 0.21), the b-wave ratio was significantly (*p* < 0.001) reduced in the I/R + Vehicle group (0.10 ± 0.07). Notably, after postischemia XFZYD administration, there was also a dose-responsive (at 1.35 g/kg/day; 0.10 ± 0.10; *p* = 0.91) and significant (at 2.7 g/kg/day; 0.33 ± 0.11; *p* = 0.003) counteraction of the ischemia-associated b-wave ratio reduction.

On the other hand, the ERG b-wave ratios of the sham eye (*n* = 9) did not significantly (*p* = 0.86) differ from those of the normal eye (*n* = 5; 1.03 ± 0.35).

### The effect of XFZYD on the density of retrograde fluorogold labeled RGCs

**Fig. 2:**
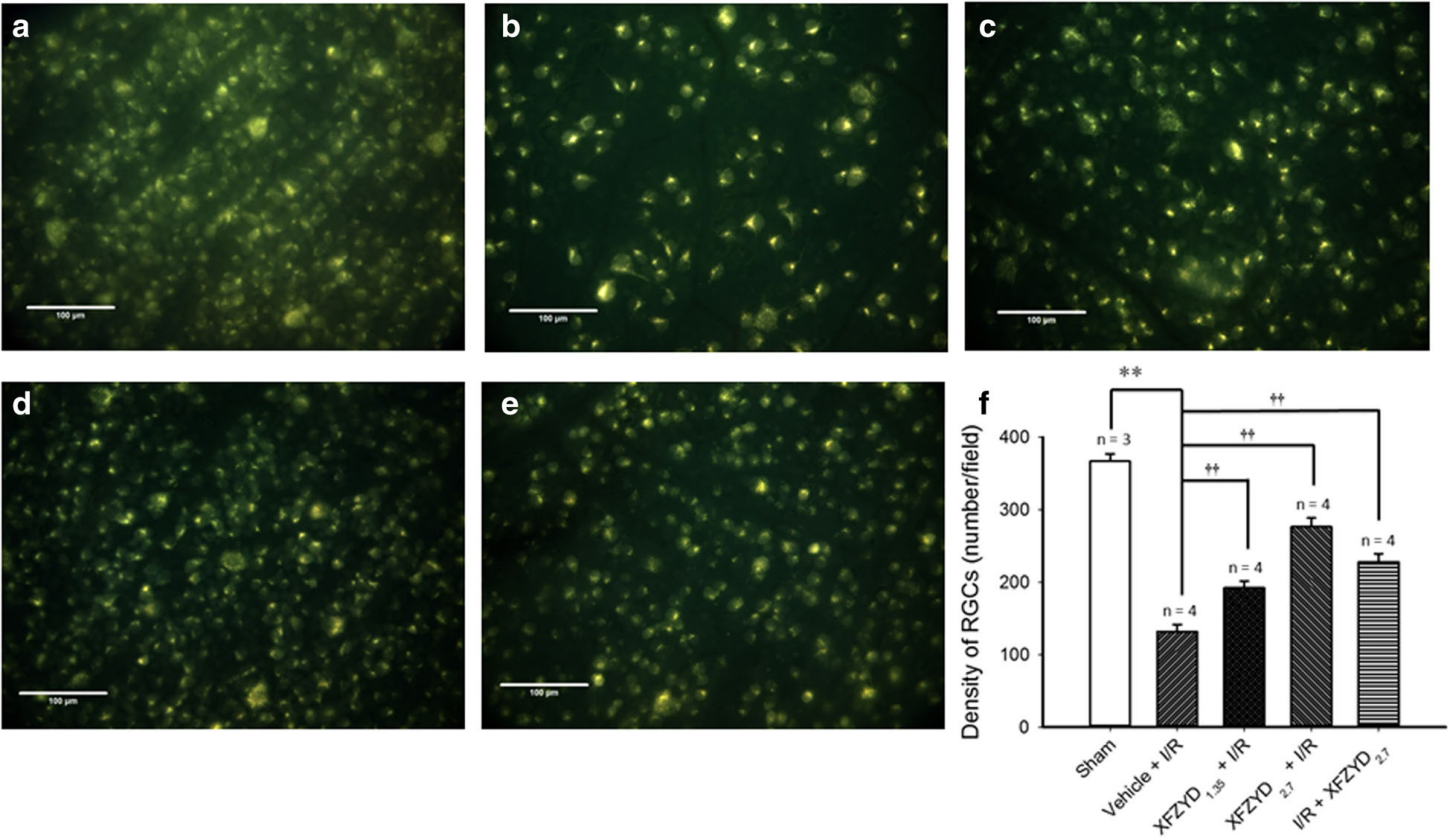
Retrograde staining with fluorogold. The micrographs revealed the density of retinal ganglion cells (RGCs) after the sham process (**a**, Sham), ischemia plus reperfusion (I/R) with preadministered vehicle (**b**, Vehicle + I/R), preadministered XFZYD at 1.35 g/kg/day (**c**, XFZYD_1.35_ + I/R), preadministered XFZYD at 2.7 g/kg/day (**d**, XFZYD_2.7_ + I/R), or postadministered XFZYD at 2.7 g/kg/day (e, I/R + XFZYD_2.7_). Quantitative analysis of the density of RGCs is shown (**f**). The mean values ± SD (*n* = 3 ~ 4) was shown for each bar. ** represents a significant difference (*p* < 0.01; Sham vs. Vehicle + I/R); †† represents a significant difference (*p* < 0.01; Vehicle + I/R vs. XFZYD_1.35_ + I/R, XFZYD_2.7_ + I/R or I/R + XFZYD_2.7_). XFZYD, Xue-Fu-Zhu-Yu decoction. Scale bars = 100 μm

As shown in Fig. 2, the density of RGCs in the sham retina (Sham, Fig. 2a and f) was 366.78 ± 10.30 cells/field. Compared to the Sham retina, the density of RGCs was significantly (*p* < 0.001) reduced to 131.83 ± 9.78 cells/field in rats subjected to retinal ischemia and preischemia administration of vehicle (Vehicle + I/R, Fig. 2b and f). Moreover, this reduction was dose-responsively and significantly (*p* < 0.001) attenuated (with a smaller influence at 1.35 g/kg/day) when the rats were subjected to retinal ischemia and preadministrated with 1.35 and 2.7 g/Kg/day XFZYD (XFZYD_1.35_ + I/R, Fig. 2c and f: 191.38 ± 9.45 cells/field; XFZYD_2.7_ + I/R, Fig. 2d and f: 276.33 ± 12.11 cells/field). Postischemia administration of 2.7 g/Kg/day XFZYD also significantly (*p* < 0.001) attenuated this ischemia-induced reduction (I/R + XFZYD_2.7_, Fig. 2e and f: 227.58 ± 11.60 cells/field). The area of one field is approximately 0.25 mm^2^.

### The influence of XFZYD on the thickness of the different retinal layers labelled with cresyl violet

**Fig. 3:**
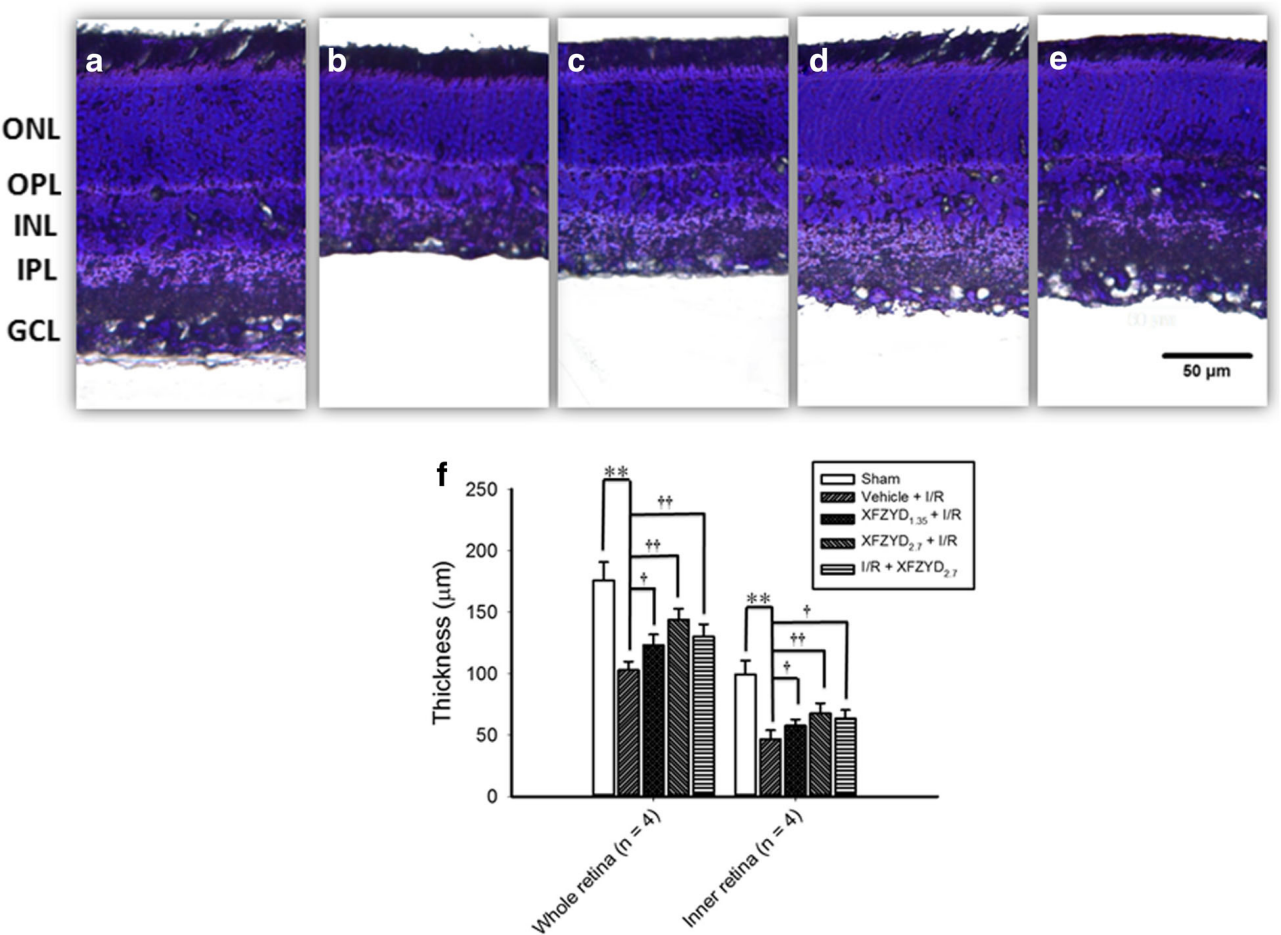
Cresyl violet staining: analysis of the thickness of various retinal layers. Sectioned retinal pieces of the identical eccentricity are shown. **a** is from a retina subjected to the sham procedure (Sham). **b** is from a retina that was given ischemia plus reperfusion (I/R) and preischemia administration of vehicle. **c**, **d**, **e** are sections from retinas that have received ischemia plus reperfusion and preischemia administration of 1.35 g/kg/day XFZYD (c, XFZYD_1.35_ + I/R), 2.7 g/kg/day XFZYD (d, XFZYD_2.7_ + I/R) or postischemia administration with 2.7 g/kg/day XFZYD (e, I/R + XFZYD_2.7_). The thickness of the different retinal layers from sectioned retinal pieces of the identical eccentricity is demonstrated. **f** The results indicate the mean values ± SD (*n* = 4). ** indicates a significant difference (*p <* 0.01) from the Sham retina. † or †† indicates a significant difference (*p <* 0.05 or *p <* 0.01) from the Vehicle + I/R retina. Abbreviations: XFZYD, Xue-Fu-Zhu-Yu decoction; ONL, outer nuclear layer; OPL, outer plexiform layer; INL, inner nuclear layer; IPL, inner plexiform layer; GCL, ganglion cell layer. Scale bar = 50 μm

Figure 3 shows the results of retinal sections obtained from the same distance (1 mm from optic nerve head) across the five groups (*n* = 4). As compared with the retina subjected to the sham procedure (Sham, Fig. 3a and f: 176.0 ± 14.81 μm for the whole retina, 99.50 ± 11.33 μm for the inner retina), following I/R and preadministrating the rats with vehicle, the thickness of the whole retina and the inner retina (Vehicle + I/R, Fig. 3b and f: 103.00 ± 6.88 μm for the whole retina, 46.75 ± 7.27 μm for the inner retina) were significantly (*p* < 0.001) reduced. Moreover, the decrease was dose-dependently (with a smaller influence at 1.35 g/kg/day) and significantly attenuated when the rats were subjected to I/R and preadministrated with 1.35 and 2.7 g/Kg/day XFZYD [XFZYD_1.35_ + I/R, Fig. 3c and f: 123.25 ± 8.62 μm for the full-thickness retina (*p* = 0.01), 57.75 ± 5.06 μm for the inner retina (*p* = 0.048); XFZYD_2.7_ + I/R, Fig. 3d and f: 144.00 ± 8.83 μm for the whole retina (*p* < 0.001), 67.75 ± 8.18 μm for the inner retina (*p* = 0.009)]. Postischemia administration of 2.7 g/Kg/day XFZYD also significantly attenuated this ischemia-induced decrease [I/R + XFZYD_2.7_, Fig. 3e and f: 130.25 ± 9.98 μm for the full-thickness retina (*p* = 0.004), 63.50 ± 6.95 μm for the inner retina (*p* = 0.016)].

### The influence of XFZYD on ChAT immunolabeling

**Fig. 4:**
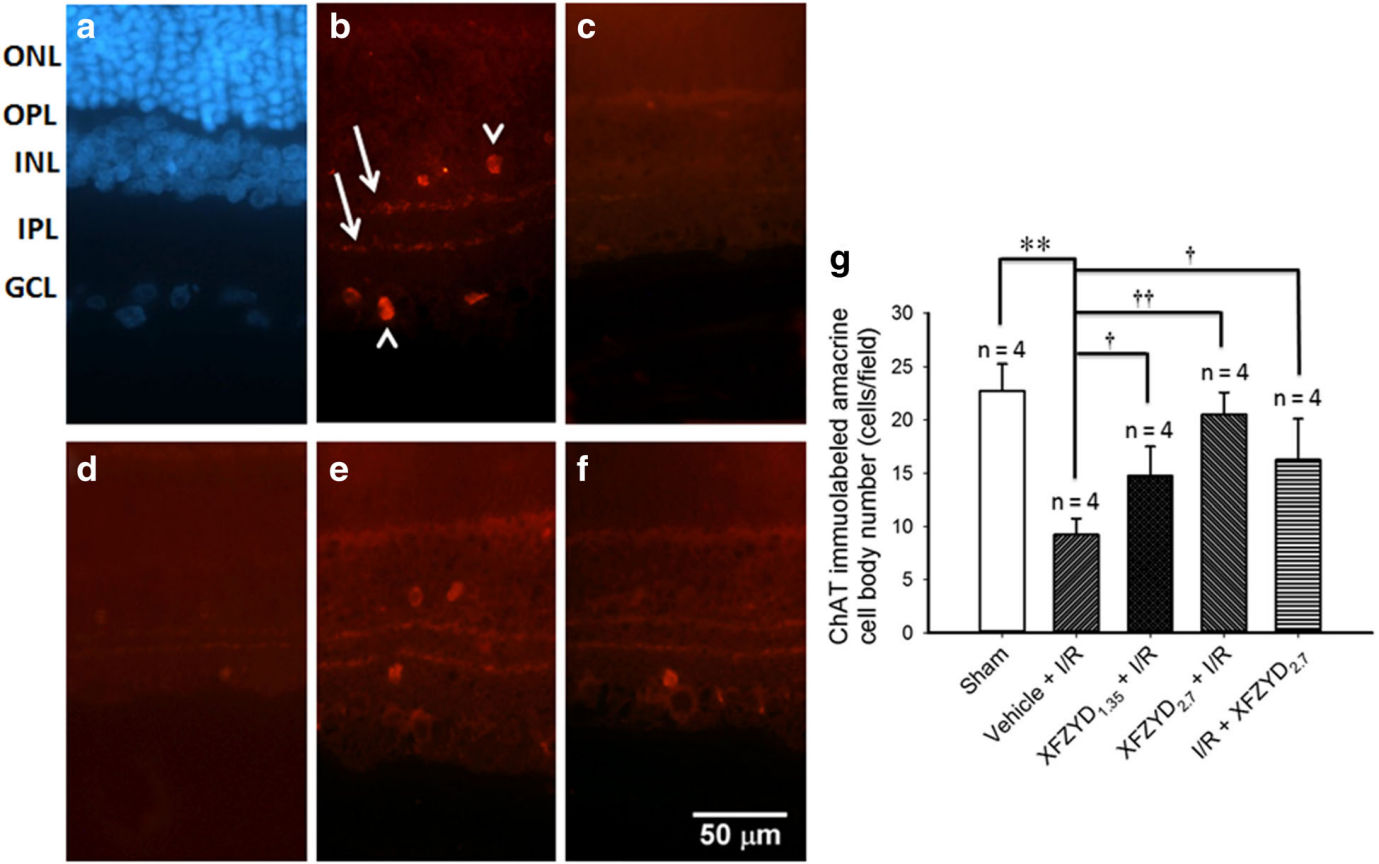
Choline acetyltransferase (ChAT, red) immunohistochemistry. **a** is the retina subjected to the sham procedure (Sham) and the cellular nucleus is labeled with 4,6-diamidine-2-phenylindole dihydrochloride (*DAPI, blue*). **b** shows the amacrine cell bodies (*Sham; arrow heads*) located in the INL and the GCL and their neuronal fibers (*arrows*); these demonstrate two distinct bands in the IPL. **c** is from the retina that received I/R and preischemia administration of vehicle; there is a drastic decrease in the IPL immunolabelling as well as the amacrine cell body number. **d**, **e**, **f** are sections from the retinas that received I/R and preischemia administration of 1.35 g/kg/day XFZYD (d, XFZYD_1.35_ + I/R), 2.7 g/kg/day XFZYD (e, XFZYD_2.7_ + I/R) or postischemia administration of 2.7 g/kg/day XFZYD (**f**, I/R + XFZYD_2.7_). Moreover, these ischemia-induced alterations were clearly and dose-dependently alleviated when the ischemic retinas were preadministrated with 1.35 and 2.7 g/Kg/day XFZYD. Postischemia administration of 2.7 g/Kg/day XFZYD also clearly attenuated these ischemia-induced alterations. Each bar represents the mean values ± SD (*n* = 4) following the sham procedure or I/R (**g**). ** indicates significance (*p* < 0.01; Sham vs. Vehicle + I/R). † or †† indicates significance (*p <* 0.05 or *p* < 0.01; Vehicle + I/R vs. XFZYD_1.35_ + I/R, XFZYD_2.7_ + I/R or I/R + XFZYD_2.7_). The area of one field is approximately 0.25 mm^2^. Abbreviations: ONL, outer nuclear layer, OPL, outer plexiform layer, INL, inner nuclear layer, IPL, inner plexiform layer, GCL, ganglion cell layer. XFZYD, Xue-Fu-Zhu-Yu decoction. Scale bar = 50 μm

As demonstrated in Fig. 4, in the retina subjected to the sham procedure (Sham, Fig. 4b), ChAT-immunopositive (red) amacrine cell bodies (arrow heads; Fig. 4g: 22.75 ± 2.50/field) in the INL and the ganglion cell layer (GCL); and their neuronal fibers formed two well-defined strata (arrows) in the inner plexiform layer. In the ischemic retina preadministrated with vehicle (Vehicle + I/R; Fig. 4c), there was a significantly (*p* < 0.001) smaller number of the ChAT-immunopositive amacrine cell bodies (Fig. 4g: 9.25 ± 1.50/field) after retinal I/R and preischemia administration of vehicle; moreover, the IPL immunoreactivity of these cells was drastically decreased. Clinically and importantly, these alterations were dose-responsively and significantly alleviated in the ischemic retinas when 1.35 and 2.7 g/Kg/day XFZYD were preadministrated (XFZYD_1.35_ + I/R, Fig. 4d and g: 14.75 ± 2.75/field, *p* = 0.013); XFZYD_2.7_ + I/R, Fig. 4e and g: 20.50 ± 2.08/field, *p* < 0.001). Postischemia administration of 2.7 g/Kg/day XFZYD (I/R + XFZYD_2.7_; Fig. 4f and g: 16.25 ± 3.86/field; *p* = 0.015) also clearly attenuated these ischemia-induced alterations. DAPI (blue, Fig 4a) was used to label the cellular nucleus in the Sham retina. The area of one field is approximately 0.25 mm^2^.

### The effect of XFZYD on GFAP or Vimentin immunolabeling

**Fig. 5:**
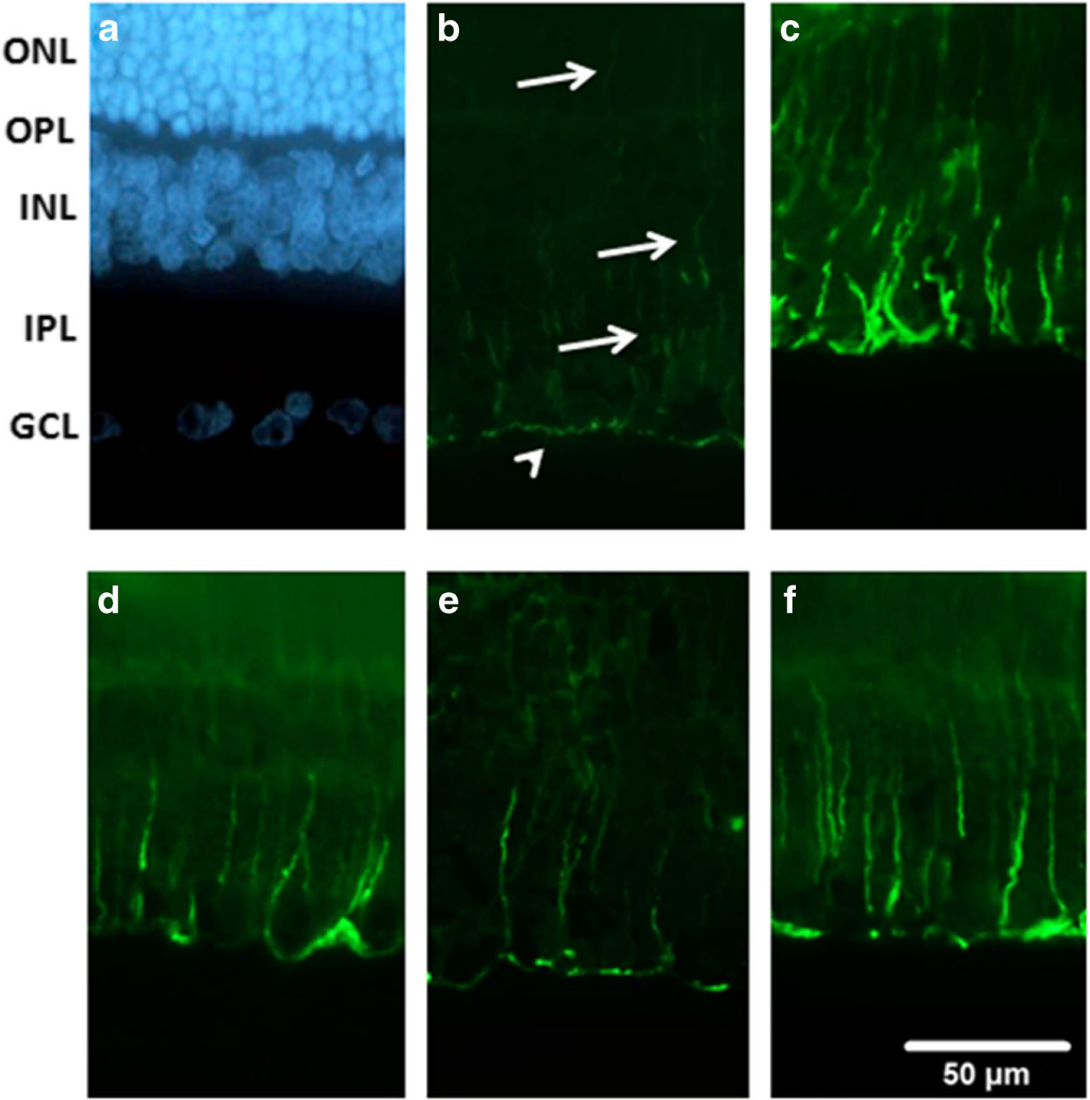
Glial fibrillary acidic protein (GFAP, green) immunohistochemistry. **a** is the cellular nucleus of the sham retina labeled with 4,6-diamidine-2-phenylindole dihydrochloride (DAPI, blue); **b** shows Müller cells labelled with GFAP immunoreactivity at the end feet (arrow head) in the GCL and at the processes in the IPL, INL and ONL (arrows). **c** shows a retina that received I/R and preischemia adminstration of vehicle; there is an increase in anti-GFAP immuolabeling. (**d**, **e**, **f**) are sections from the retinas that received I/R and preischemia administration of 1.35 g/kg/day XFZYD (**d**, XFZYD_1.35_ + I/R), 2.7 g/kg/day XFZYD (**e**, XFZYD_2.7_ + I/R) or postischemia administration of 2.7 g/kg/day XFZYD (**f**, I/R + XFZYD_2.7_). Moreover, this ischemia-associated alteration was obviously and dose-dependently mitigated as ischemic retinas were preadministrated with 1.35 and 2.7 g/Kg/day XFZYD. Postischemia administration of 2.7 g/Kg/day XFZYD also obviously mitigated this ischemia-induced alteration. ONL: outer nuclear layer, OPL: outer plexiform layer, INL: inner nuclear layer, IPL: inner plexiform layer, GCL: ganglion cell layer. Scale bar = 50 μm

As demonstrated in Fig. 5, in the Sham retina (Fig. 5b), the Müller cells could be identified using the GFAP immunolabelings at the end feet (arrow head) in the GCL and at the processes that extended into the IPL, INL and ONL (arrows). In the ischemic retina preadministrated with vehicle (Vehicle + I/R, Fig. 5c), the anti-GFAP immuolabeling was enhanced. Moreover, this alteration was clearly and dose-dependently mitigated in the ischemic retinas preadministrated with 1.35 and 2.7 g/Kg/day XFZYD (XFZYD_1.35_ + I/R, Fig. 5d; XFZYD_2.7_ + I/R, Fig. 5e). Postischemia administration of 2.7 g/Kg/day XFZYD (I/R + XFZYD_2.7_, Fig. 5f) also clearly attenuated this ischemia-induced alteration.

**Fig. 6:**
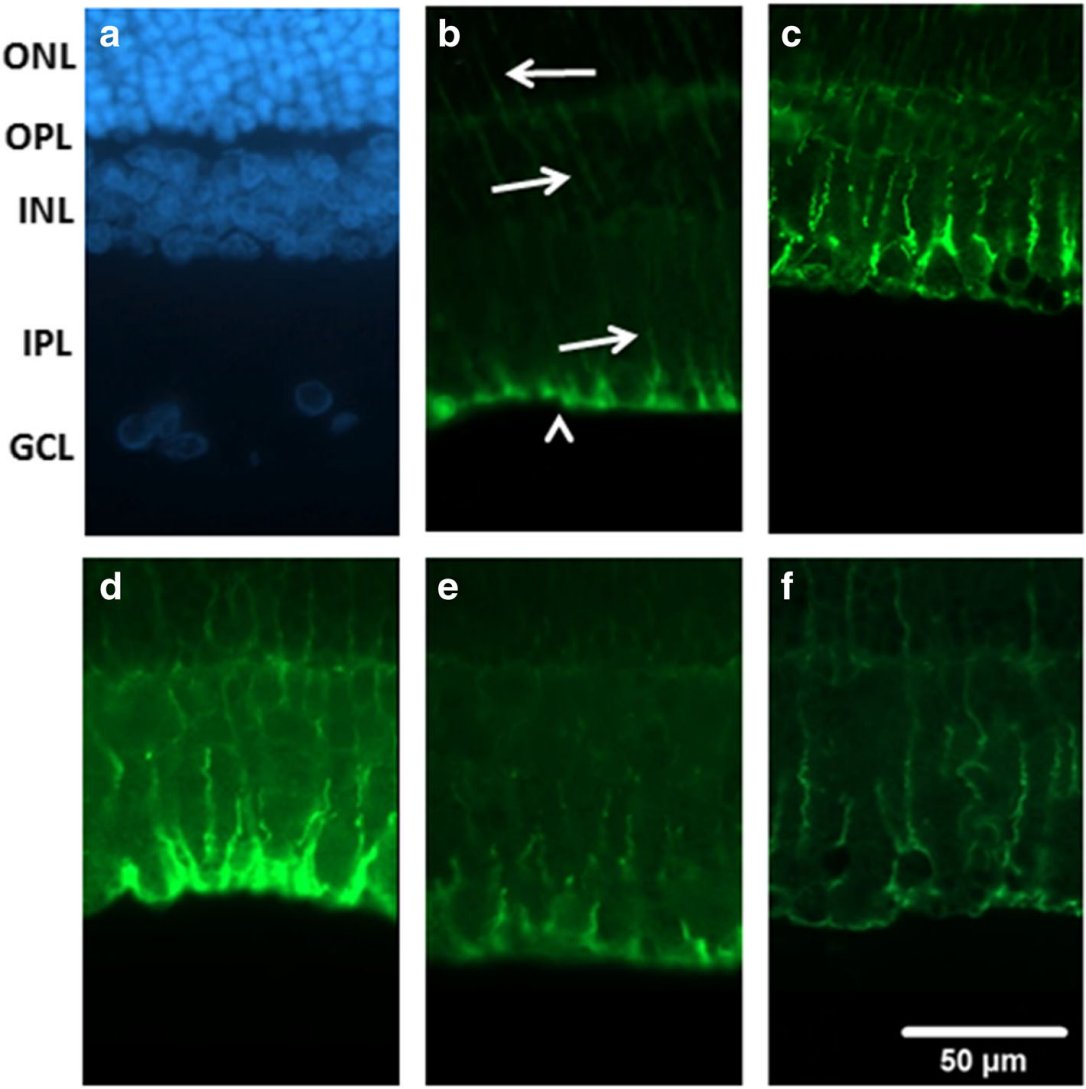
Vimentin (green) immunohistochemistry. **a** shows the cellular nucleus of the sham retina labeled with 4,6-diamidine-2-phenylindole dihydrochloride (*DAPI, blue*). **b** shows a sham retina with the vimentin immunoreactivity at the end feet (*arrow heads*) in the GCL and at the processes in the IPL, INL and ONL (*arrows*). **c** shows a retina that received I/R and preischemia adminstration of vehicle; in contrast to the sham retina, the anti-vimentin immuolabeling has increased. (**d**, **e**, **f**) are sections from retinas that received I/R and preischemia administration of 1.35 g/kg/day XFZYD (d, XFZYD_1.35_ + I/R), 2.7 g/kg/day XFZYD (**e**, XFZYD_2.7_ + I/R) or postischemia administration of 2.7 g/kg/day XFZYD (**f**, I/R + XFZYD_2.7_). Moreover, this ischemia-induced alteration is clearly and dose-dependently reduced when the ischemic retina is preadministrated with 1.35 and 2.7 g/Kg/day XFZYD. Postischemia administration of 2.7 g/Kg/day XFZYD also clearly attenuated this ischemia-induced alteration. ONL: outer nuclear layer, OPL: outer plexiform layer, INL: inner nuclear layer, IPL: inner plexiform layer, GCL: ganglion cell layer. Scale bar = 50 μm

In comparison to the control retina (Sham, Fig 6b), anti-vimentin immunoreactivity was also enhanced after retinal ischemia and preischemia administration of vehicle (Vehicle + I/R, Fig 6c). This enhancement was drastically and in a dose-responsive manner counteracted by preischemia administration of 1.35 and 2.7 g/Kg/day XFZYD (XFZYD_1.35_ + I/R, Fig. 6d; XFZYD_2.7_ + I/R, Fig 6e). Postischemia administration of 2.7 g/Kg/day XFZYD (I/R + XFZYD_2.7_, Fig 6f) also considerably obliterated this ischemia-induced alteration. DAPI (blue; Figs. 5a and 6a) was used to label the cellular nucleus of the Sham retina.

### The influence of XFZYD on the mRNA levels of VEGF, HIF-1α, PKM2 and RBP2 in the retina

**Fig. 7:**
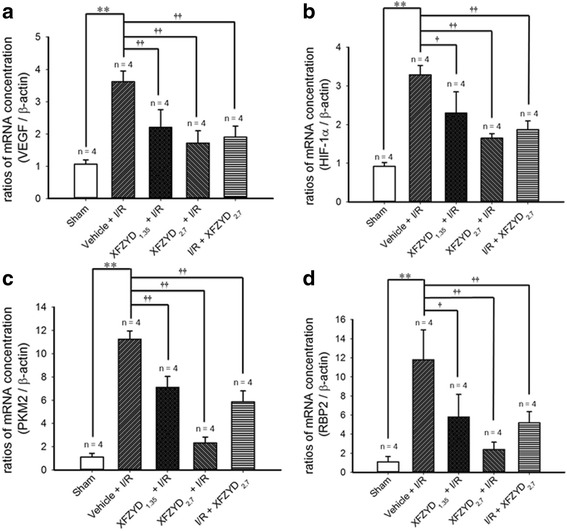
The ratios of the mRNA concentrations of VEGF, HIF-1α, PKM2 and RBP2 relative to that of β-actin. Total mRNA was extracted from the sham retinas or the ischemic retinas pretreated with vehicle, a low dose XFZYD (1.35 g/Kg/day), a high dose XFZYD (2.7 g/kg/day), or posttreated with a high dose XFZUD. The mRNA concentration ratios of VEGF (**a**), HIF-1α (**b**), PKM2 (**c**) and RBP2 (**d**) relative to β-actin were calculated. The mean values ± SD (*n* = 4) was shown for each bar. ** (*p* < 0.01) represents significance (Sham vs. Vehicle + I/R). † (*p* < 0.05) or †† (*p* < 0.01) represents significance (Vehicle + I/R vs. XFZYD_1.35_ + I/R, XFZYD_2.7_ + I/R, or I/R + XFZYD_2.7_). VEGF: vascular endothelial growth factor, HIF-1α: hypoxia inducible factor 1α, PKM2: pyruvate kinase M2, RBP2: retinoblastoma-binding protein 2

As demonstrated in Fig. 7 (*n* = 4), in contrast with the control retina (Sham; VEGF = 1.06 ± 0.13, HIF-1α = 0.92 ± 0.10, PKM2 = 1.10 ± 0.33, RBP2 = 1.09 ± 0.56), the ratios for VEGF (A), HIF-1α (B), PKM2 (C) and RBP2 (D) in the ischemic retina preadministrated with vehicle (Vehicle + I/R; VEGF = 3.62 ± 0.33, HIF-1α = 3.29 ± 0.24, PKM2 = 11.25 ± 0.71, RBP2 = 11.80 ± 3.14) were significantly (*p* < 0.001) elevated. Moreover, this enhancement was dose-dependently (with a smaller influence at 1.35 g/kg/day) and significantly [XFZYD_1.35_ + I/R (VEGF = 2.2 ± 0.55, *p* = 0.005; HIF-1α = 2.30 ± 0.55, *p* = 0.016; PKM2 = 7.11 ± 0.93, *p* < 0.001; RBP2 = 5.80 ± 2.37, *p* = 0.023); XFZYD_2.7_ + I/R (VEGF = 1.7 ± 0.38, HIF-1α = 1.65 ± 0.12, PKM2 = 2.32 ± 0.51, *p* < 0.001; RBP2 = 2.39 ± 0.77, *p* = 0.001)] counteracted in ischemic retinas preadministrated with 1.35 and 2.7 g/Kg/day XFZYD. Postischemia administration of 2.7 g/Kg/day XFZYD also significantly [I/R + XFZYD_2.7_ (VEGF = 1.90 ± 0.34; HIF-1α = 1.87 ± 0.22; PKM2 = 5.86 ± 0.95, *p* < 0.001; RBP2 = 5.19 ± 1.17, *p* = 0.008)] mitigated this ischemia-induced increase.

In addition, when the retinal mRNA concentrations of VEGF, HIF-1α, PKM2 and RBP2 were compared, no significant difference existed between the sham eye and the normal eye (*n* = 4; VEGF = 1.02 ± 0.11, *p* = 0.36; HIF-1α = 1.00 ± 0.03, *p* = 0.20; PKM2 = 1.02 ± 0.13, *p* = 0.36; RBP2 = 1.00 ± 0.04, *p* = 0.39).

### The influence of XFZYD on the concentrations of various proteins in the retina

**Fig. 8:**
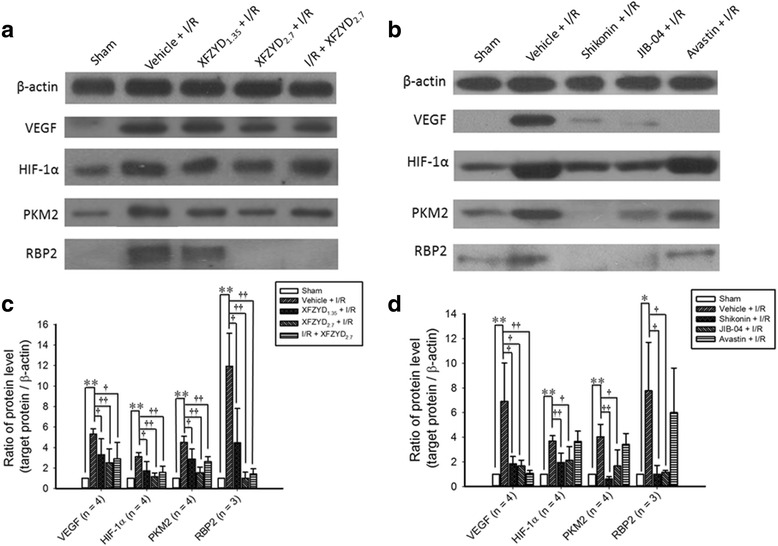
The ratios of the protein concentrations of VEGF, HIF-1α, PKM2 and RBP2 relative to β-Actin. **a** and **b** showed the Western blotting for expressions of β-actin, VEGF, HIF-1α, PKM2 and RBP2. In figure a, Lane 1 is a retina that received the sham procedure (Sham); Lane 2 is the vehicle-preadministered ischemic retina (Vehicle + I/R); Lanes 3 ~ 5 are from retinas that received ischemia plus reperfusion and were preadministrated with 1.35 g/kg/day XFZYD (XFZYD_1.35_ + I/R), 2.7 g/kg/day XFZYD (XFZYD_2.7_ + I/R) or postadministrated with 2.7 g/kg/day XFZYD (I/R + XFZYD_2.7_). In figure b, Lane 1 is from a sham retina (Control); Lane 2 is the vehicle-preadministered ischemic retina (Vehicle + I/R); Lanes 3 ~ 5 are from retinas that received ischemia plus reperfusion and were preadministrated with 4 μM Shikonin (PKM2 inhibitor), 10 μM JIB-04 (RBP2 inhibitor) and 100 mg/4 ml Avastin (anti-VEGF). (c and d) showed the protein levels of VEGF (*n* = 4), HIF-1α (*n* = 4), PKM2 (*n* = 4) and RBP2 (*n* = 3) divided by the level of β-actin. */** or †/†† indicates significance (*p* < 0.05/*p* < 0.01; pictures (**c** and **d**): Sham vs. Vehicle + I/R) or significance (*p* < 0.05/*p* < 0.01; picture **c**: Vehicle + I/R vs. XFZYD_1.35_ + I/R, XFZYD_2.7_ + I/R, or I/R + XFZYD_2.7_; Picture d: Vehicle + I/R vs. Shikonin + I/R, JIB-04 + I/R, or Avastin + I/R). Each bar indicates the mean values ± SD. VEGF: vascular endothelial growth factor, HIF-1α: hypoxia inducible factor 1α, PKM2: pyruvate kinase M2, RBP2: retinoblastoma-binding protein 2

As demonstrated in Fig. 8a and c (*n* = 4 for VEGF, HIF-1α and PKM2; *n* = 3 for RBP2), in contrast to the control retina (Sham; VEGF, HIF-1α, PKM2 and RBP2 = 1.00), after I/R and preischemia administration of vehicle (VEGF = 5.31 ± 0.53, HIF-1α = 3.10 ± 0.40, PKM2 = 4.51 ± 0.60, RBP2 = 11.92 ± 3.22), there was a significant (*n* = 4, VEGF, HIF-1α and PKM2, *p* < 0.001; *n* = 3, RBP2, *p* = 0.004) increase in the protein ratios for VEGF, HIF-1α, PKM2 and RBP2. Moreover, this enhancement was dose-dependently (with a smaller influence at 1.35 g/kg/day) and significantly [XFZYD_1.35_ + I/R (VEGF = 3.31 ± 1.54, *p* = 0.049; HIF-1α = 1.71 ± 0.92, *p* = 0.032; PKM2 = 2.88 ± 0.98, *p* = 0.022; RBP2 = 4.46 ± 3.35, *p* = 0.049; XFZYD_2.7_ + I/R (VEGF = 2.52 ± 1.35, *p* = 0.008; HIF-1α = 1.16 ± 0.36, PKM2 = 1.56 ± 0.53, *p* < 0.001; RBP2 = 1.00 ± 0.59, *p* = 0.004)] mitigated when the ischemic retinas were preadministrated with 1.35 and 2.7 g/Kg/day XFZYD. Postischemia administration of 2.7 g/Kg/day XFZYD also significantly (I/R + XFZYD_2.7_: VEGF = 2.91 ± 1.59, *p* = 0.029; HIF-1α = 1.57 ± 0.61, *p* = 0.006; PKM2 = 2.62 ± 0.49, *p* = 0.002; RBP2 = 1.42 ± 0.52, *p* = 0.005) attenuated this ischemia-induced increase. In addition (Fig. 8b and d), significant attenuation of the ischemia-induced increase in the ratio of VEGF (Vehicle = 6.92 ± 1.55; Shikonin = 1.84 ± 0.60, *p* = 0.018; JIB-04 = 1.68 ± 0.46, *p* = 0.016; Avastin = 1.08 ± 0.23, *p* = 0.01), HIF-1α (Vehicle = 3.69 ± 0.22; Shikonin = 1.95 ± 0.76, *p* = 0.007; JIB-04 = 2.14 ± 1.11, *p* = 0.04; Avastin = 3.65 ± 0.84, *p* = 0.942), PKM2 (Vehicle = 4.04 ± 0.50; Shikonin = 0.61 ± 0.19, *p* < 0.001; JIB-04 = 1.67 ± 1.31, *p* = 0.028; Avastin = 3.42 ± 0.88, *p* = 0.390), and RBP2 (Vehicle = 7.77 ± 2.27; Shikonin = 1.00 ± 0.71, *p* = 0.043; JIB-04 = 1.16 ± 0.14, *p* = 0.044; Avastin = 6.00 ± 3.61, *p* = 0.597) was significantly attenuated by preischemia administration of various inhibitors/antibodies 4 μM Shikonin (PKM2 inhibitor), 10 μM JIB-04 (RBP2 inhibitor) and 100 mg/4 ml Avastin (VEGF antibody), but this did not occur with vehicle.

In addition, when the retinal protein concentrations of VEGF, HIF-1α, PKM2 and RBP2 were compared, no significant difference existed between the sham eye and the normal eye (*n* = 4; VEGF = 0.94 ± 0.01, *p* = 0.46; HIF-1α = 0.92 ± 0.02, *p* = 0.26; PKM2 = 0.46 ± 0.01, *p* = 0.10; RBP2 = 1.22 ± 0.09, *p* = 0.37).

## Discussion

Ischemia is widely accepted to be involved in vision-threatening retinal disorders such as, proliferative diabetic retinopathy (PDR) [26], wet age-related macular degeneration (wAMD) [27] and central/branch retinal vein or artery occlusion [4]. Furthermore, although, anti-VEGF antibodies are clinically utilized world-wide, these are not completely effective when treating these conditions [28]. Moreover, disappointing visual results often occur in patients who have been treated with anti-VEGF drugs, even though the vitreous/subretinal blood and cystoid macular edema have been effectively treated. KDM5A (JARID1A; RBP2) was originally identified as a factor that interacts with the RB gene product [29, 30]. In mammals, there are four PK isoforms including PKM2, which has been found in the retina, an extension of the central nervous system, and the brain [31–33]. Avastin is widely known to target all VEGF-A isoforms; isotype 165 is most abundant and biologically active in the eye. The mode of action of another recently launched anti-VEGF aflibercept is involved in VEGF-A, VEGF-B and placental growth factor. As mentioned in the Introduction, PKM2 and RBP2 co-activate HIF-1α, which further triggers VEGF secretion and induces possible subsequent angiogenesis in the ischemic/hypoxic conditions [6, 7, 34–36]. However, there remained a need to investigate whether PKM2 might be an upstream factor that links with the activation of RBP2.

Furthermore, there is also a clinical necessity to identify anti-ischemic agents that possess novel pharmaceutical mechanisms, such as blocking RBP2 and PKM2. The enhanced mRNA/protein concentrations of RBP2 and PKM2, as well as the consequential stimulation of HIF-1α and VEGF mRNA/protein levels as measured during the present investigation are clearly associated with retinal ischemia and, possibly, wAMD. As also mentioned in the Introduction, XFZYD has been found to potentiate recombinant tPA-mediated neuroprotection against ischemic stroke in the rats [11] and act as an antioxidant. It is also well known that *Radix paeoniae rubra, Rhizoma chuanxi*o*ng, Semen pruni persicae*, and *Carthamus tinctorius* have an ability to ameliorate the blood circulation and clean up the blood clot as mentioned [11, 13, 14, 16–18, 37]*.* If the present study helps to identify novel protective mechanisms, CJDHW could offer novel means of coping with the various retinal ischemic diseases mentioned earlier that threaten vision.

GFAP and Vimentin immunoreactivity were enhanced in Müller cells following retinal I/R [3], and this is supported by the current result (Figs. 5c and 6c). Dysfunctional Müller cells produce a negative influence on normal ERG b-wave [3, 38, 39], which is also found in this present results (Fig. 1). When retinas are subjected to ischemia and preadministrated with vehicle, enhanced vimentin/GFAP immunoreactivity was found to be present simultaneously with the reduction in b-wave amplitude. Notably, these ischemic changes were mitigated when there was preischemia or postischemia administration of XFZUD to the experimental rats.

After retinal I/R, glutamate receptor-containing neurons, for example RGCs and amacrines, which are present in the inner retina are widely accepted to be liable to injury [1, 3, 24, 25, 39]. In the present study, following I/R, it was confirmed that the number of RGCs was significantly decreased (Fig. 2b and f) and retinal thickness, especially inner retinal thickness, was significantly reduced (Fig. 3b and f). In addition, the result of ChAT immunohistochemistry showed that the number of the amacrine cell bodies was significantly fewer (Fig. 4c and g). Of particular clinical importance, our findings also reveal that preischemia or postischemia administration of XFZYD is able to bring about a significant counteraction of these ischemic features, namely an attenuation of the ischemia-induced decrease in the number of the RGCs (Fig. 2c~f), an alleviation of the ischemia-associated decrease in the inner/whole retina thickness (Fig. 3c~f), as well as mitigation of the ischemia-related reduction in amacrine cell bodies (Fig. 4d~g).

Previous studies have found that HIF-1α plays a vital part in the activation of hypoxia related VEGF release [6, 35, 40]. Moreover, Luo et al. [40] found that PKM2 reacts with HIF-1α in a physical and functional manner to trigger the linkage of HIF-1α with target genes, including coactivators and gene transcription. Moreover, Qi et al. [35] indicated that the RBP2 protein is able to trigger HIF-1α upregulation and, which in turn stimulates VEGF release. In the present study, it has been verified that the mRNA/protein levels of VEGF, HIF-1α, PKM2 and RBP2 (Figs 7 and 8) are increased in the ischemic retina. As shown by Fig. 8, the effect of JIB-04 on the mean protein levels of RBP2 relative to effect of XFZUD on the mean protein levels of RBP2 was 1.16 to 1.00 (*p* = 0.668). As discussed in the beginning, PKM2 and RBP2 co-activate HIF-1α and then stimulates VEGF release [35, 36]. Not inconsistently with the present result, preischemia or postischemia administration of XFZYD significantly counteracted these ischemia-induced increases (Fig. 8). Taken together, this implies that XFZYD might be able to prevent or protect the defined retinal ischemic changes via a synergistic inhibition of RBP2 and PKM2 as well as by the further down-regulation of HIF-1α and a subsequent reduction in VEGF-A secretion.

The Rhesus monkey studies [41, 42] have indicated that CRAO of up to 240 min leads to disastrous permanent retinal injury. BRAO eyes that have poorer presenting corrected vision are unlikely to spontaneously improve in the vision and this could result in persistent retinal injury [43, 44]. Topical brimonidine and somatostatin had been shown to act as neuroprotectants and seem to attenuate morbidity due to background diabetic retinopathy (BDR) as well as any deterioration of BDR into PDR [45]. As mentioned earlier, hypoxia, mediated by HIF-1α, both aggravates cellular injury and inflammation as well as plays an important role in angiogenesis [46]. The increased levels of HIF-1α further stimulates the overexpression of VEGF and VEGFR [47], which in turn promotes endothelium growth and new vessel formation, namely angiogenesis [11]. A recent report has indicated that XFZYD significantly reduces the level of HIF-1α and, as a consequence, VEGF [12]. In addition to the decoction’s antiangiogenic and protective effects, XFZYD would appear to have a potentially important role in preventive medicine, while at the same time providing a relevant and important pharmaceutical effect when one has a familial morbidity or have suffered from previous vision-threatening retinal ischemic disorders such as, wAMD, DR, glaucoma, CRAO, BRAO, CRVO or BRVO.

## Conclusion

The present study has clearly demonstrated various retinal ischemic changes, namely a significant decrease in the amplitudes of ERG b-wave (indexing Müller and bipolar cells), a significant less numerous RGCs, a significantly reduced inner/whole retinal thickness, and a significant loss of the ChAT immunolabeling amacrine cells as well as an obvious enhancement in GFAP/Vimentin immunolabeling (indexing Müller cells). In addition, significant upregulation of the mRNA/protein levels of VEGF, HIF-1α, PKM2 and RBP2 were also found in the ischemic retina. Importantly, the harmful influences of these aspects of ischemia were alleviated in a dose responsive and significant manner when XFZYD was applied for seven consecutive days before or after retinal ischemia. In particular, the increases in the ischemia-associated VEGF, HIF-1α, PKM2 and RBP2 increases were blunted by XFZYD. When our findings are taken as a whole, XFZYD would seem to have a preventive or protective effect on retinal ischemia via the downregulation of HIF-1α expression and a reduction in VEGF secretion; this ocurrs via an inhibition of RBP2 and PKM2.

## Additional file

Additional file 1: XFZUD No. of rats. (PDF 158 kb)

## Abbreviations

AMD: age-related macular degeneration
ANOVA: analysis of variance
ARRIVE: *Animal* Research: Reporting of In Vivo Experiments
BCVA: best corrected visual acuity.
BRAO: branch retinal artery occlusion
BRVO: branch retinal vein occlusion
cDNA: complementary DNA
ChAT: choline acetyltransferase
CHGH: Cheng Hsin General Hospital
CHIACUC: Institutional Animal Care and Use Committee at CHGH
CRAO: central retinal artery occlusion
CRVO: central retinal vein occlusion
Ct: cycle threshold
DAPI: 4,6-diamidine-2-phenylindole dihydrochloride
ERG: electroretinogram,
FITC: fluorescein isothiocyanate
GCL: ganglion cell layer
GFAP: glial fibrillary acidic protein
HIF-1α: hypoxia-inducible factor-1α
HIOP: high intraocular pressure
HSYA: hydroxysafflor yellow A
I/R: ischemia/reperfusion
INL: inner nuclear layer
IPL: inner plexiform layer
PBS: phosphate-buffered saline
PCR: polymerase chain reaction
PDR: proliferative diabetic retinopathy
PKM2: pyruvate kinase M2
*PVDF*: polyvinylidene difluoride
RBP2: retinoblastoma-binding protein 2
RGCs: retinal ganglion cells
RPE: retinal pigment epithelium
SD: standard deviation
tPA: tissue plasminogen activator
VEGF: vascular endothelium growth factor
wAMD: wet age-related macular degeneration
XFZYD: Xue-Fu-Zhu-Yu decoction
