# Supplementary material for: Xue-fu-Zhu-Yu decoction protects rats against retinal ischemia by downregulation of HIF-1α and VEGF via inhibition of RBP2 and PKM2
Source: BMC Complement Altern Med. 2017 Jul 14;17:365. doi: 10.1186/s12906-017-1857-2 (PMC5513111; doi:10.1186/s12906-017-1857-2)
Supplement: Additional file 1: — XFZUD No. of rats. (PDF 158 kb) [file 12906_2017_1857_MOESM1_ESM.pdf]

Gr/M	Nor	Sham	Veh + I/R	I/R + Veh	XF$_{1.35}$ + I/R	XF$_{2.7}$ + I/R	I/R + XF$_{1.35}$	I/R + XF$_{2.7}$	Total*
ERG	(5)†	(9)†	(6)†	5	(4)†	(8)†	6	(6)†	11
PHX	-	4	4	-	4	4	-	4	20
FG	4	4(3)††	4	-	4	4	-	4	24(23)††
PCR	4	4	4	-	4	4	-	4	24
WB	4	4	4	-	4	4	-	4	24
Ab	=	=	=	-	I/R + Avastin (n=4)	I/R + Shikonin (n=4)	I/R + JIB-04 (n=4)§		12

*The number of the rats used in this XFZUD (Xue-Fu-Zhu-Yu Decoction) study is **120** (=11+20+24+24+24+12+**5**), including **5** animals that died during the induction of pressure-induced retinal ischemia.

†After ERG recordings, among all groups except the number of the rats (5+6=11) in I/R + Veh (n=5) and I/R + XF$_{1.35}$ groups (n=6), the other rats were preserved for the following procedures, namely PHX, FG, rtPCR and WB.

††One animal died during the FG procedure.

§RBP2 western blot was ill-defined in one rat [I/R + JIB-04 (n=4-1=3)].

Abbreviations: Gr, group; M, methods;; Nor, normal; I/R, ischemia/reperfusion; Veh, vehicle; XF$_{1.35}$, XFZUD 1.35 mg/Kg; XF$_{2.7}$, XFZUD 2.7 mg/Kg; ERG, electroretinogram; PHX, pathohistology (e.g. CV, ChAT & vimentin/GFAP labelling); FG, flurogold; CV, cresyl violet; ChAT, choline acetyl transferase; GFAP, glial fibrillary acidic protein; rtPCR, real-time polymerase chain reaction; WB, western blot; Ab, antibody.
